# DNA structural properties of DNA binding sites for 21 transcription factors in the mycobacterial genome

**DOI:** 10.3389/fcimb.2023.1147544

**Published:** 2023-06-16

**Authors:** Upalabdha Dey, Kaushika Olymon, Anikesh Banik, Eshan Abbas, Venkata Rajesh Yella, Aditya Kumar

**Affiliations:** ^1^ Department of Molecular Biology and Biotechnology, Tezpur University, Tezpur, India; ^2^ Department of Biotechnology, Koneru Lakshmaiah Education Foundation, Guntur, India

**Keywords:** *Mycobacterium tuberculosis*, virulence-associated transcription factor, DNA shape, DNA flexibility, DNA curvature

## Abstract

*Mycobacterium tuberculosis*, the causative agent of tuberculosis, has evolved over time into a multidrug resistance strain that poses a serious global pandemic health threat. The ability to survive and remain dormant within the host macrophage relies on multiple transcription factors contributing to virulence. To date, very limited structural insights from crystallographic and NMR studies are available for TFs and TF–DNA binding events. Understanding the role of DNA structure in TF binding is critical to deciphering MTB pathogenicity and has yet to be resolved at the genome scale. In this work, we analyzed the compositional and conformational preference of 21 mycobacterial TFs, evident at their DNA binding sites, in local and global scales. Results suggest that most TFs prefer binding to genomic regions characterized by unique DNA structural signatures, namely, high electrostatic potential, narrow minor grooves, high propeller twist, helical twist, intrinsic curvature, and DNA rigidity compared to the flanking sequences. Additionally, preference for specific trinucleotide motifs, with clear periodic signals of tetranucleotide motifs, are observed in the vicinity of the TF–DNA interactions. Altogether, our study reports nuanced DNA shape and structural preferences of 21 TFs.

## Introduction

Tuberculosis has emerged as a global health crisis in recent decades. A total of 10.6 million people contracted tuberculosis in 2021, a 4.5% rise from the previous year’s projection of 10.1 million cases (Global tuberculosis report 2022 by WHO). *Mycobacterium tuberculosis* H37Rv, the etiological pathogen, is a very successful and formidable microbe because of its resilient cell wall, its ability to enter a latent state when it is deemed appropriate, and its arsenal of tricks to evade the body’s immune system. It has evolved into a multidrug resistant strain throughout time, posing a serious public health threat (Mukhopadhyay et al., 2012). Research into the molecular underpinnings of pathogenic potential, virulence, and resilience of mycobacteria has made significant advances in the last decade. Most of these virulence factors are part of the cell surface proteins, signaling pathways, lipid pathways, regulators, and those involved in survival (Forrellad et al., 2013). The pathogen’s adaptability to the local environment during TB disease depends on the highly coordinated regulation of gene expression orchestrated by a single RNA polymerase, ~13 sigma factors, and >200 DNA-binding proteins.

The MTB RNA polymerase is a ∼400-kDa multiprotein complex with a catalytic core enzyme, (subunit composition α2ββ′ω), which executes the transcription process of all cellular RNAs (Kouba et al., 2020). The σ (sigma) factors that are necessary to initiate transcription in a way unique to a promoter form the holoenzyme after binding to the catalytic core of the RNAP (Davis et al., 2014). The RNAP holoenzyme binds the DNA promoter through the sequence-specific recognition of the −35 and the −10 upstream elements. The performance of RNAP is fine-tuned by sigma factors, transcription factors (TFs), regulatory DNA sequences, and the GC content of the promoter region (Herrera-Asmat et al., 2017). Different promoters have different transcriptional yields because the speed of the individual steps leading to the open complex are regulated by the promoter region (Chen et al., 2021). *M. tuberculosis* encodes 13 sigma factors, out of which it has been revealed through mutant screening and complementation that (Agarwal et al., 2007) sigD and sigE are mostly responsible for the bacterium’s ability to respond to different environments, and thus play a key role in the organism’s virulence (Mukhopadhyay et al., 2012). MTB has unique and synchronized regulatory attributes that are under the control of approximately 214 TFs coupled with RNAP-binding proteins (i.e., CarD, RbpA, and Nus) and an essential two-component system (TCS) that regulates host gene expression under various conditions. Gene knockdown, chromatin immunoprecipitation, upregulation, and several *in silico* analyses have been applied to characterize certain MTB TBs (Rustad et al., 2014). Cell viability in the face of environmental change requires a transcription process that can adapt quickly and effortlessly to sustain physiologically relevant quantities of TFs. All such proteins change the transcriptional architecture by interacting with DNA and various proteins along with chemical messengers, thus transforming recurrent signals and inputs into synchronized downstream gene expressions (Minch et al., 2015). The roles of several TBs in the survival of MTB have been demonstrated under a wide range of host-induced stresses, including cold shock (Shires and Steyn, 2001), heat shock (Manganelli et al., 1999; Stewart et al., 2002), nutrient starvation (Rodriguez and Smith, 2003; Canneva et al., 2007), reactive nitrogen intermediates (Voskuil et al., 2003), and hypoxia (Park et al., 2003). Bacilli respond to these stresses by inducing transcriptional variation, which causes them to activate stress response genes, alter their metabolism, and secrete virulence proteins, in order to survive the hostile response of the host immune system (Kumar et al., 2022). DosR, MprA, TcrA, TrcR, TcrX, SigH, SigB, SigE, and PhoP are some of the best-studied TCSs and sigma factors in Mtb, and they all play important roles in mediating viability under different stresses. A hallmark of MTB’s response to hypoxia and nitrosative stress is the expression of DevR and its regulon genes. In addition to their roles in virulence, phosphate absorption, and aerobic respiration, PhoP, MtrA, MprA, and KstR/Rv3574 have been implicated in the control of WhiB proteins and the complex lipid biosynthetic pathway, the stress response of the cell envelope, the modulation of sigma factors, and survival (Nandi et al., 2019). Although transition metals like copper are important for the growth and development of MTB, excessive use of it becomes toxic. CsoR/Rv0967, is an important copper-sensitive repressor, having a noble DNA binding domain and a key role in copper homeostasis as well as pathogenicity of MTB (Samanovic et al., 2012). The genome of *M. tuberculosis* encompasses seven members of the WhiB superfamily that have putative roles in sensing nitric oxide and oxygen. The expression of these genes tends to increase in MDR clinical specimens (Miotto et al., 2022). Furthermore, Rv0023 and Rv0465c/RamB, classified as a member of the xenobiotic response element (XRE), are important modulators of transcription, where the former is implicated in NADH/NAD+ regulation and also acts a negative regulator for WhiB5/Rv0022c while the latter plays a significant role in propionate metabolism in MTB (Gupta et al., 2020). Some of the MTB TFs, including Rv3830c, Rv0767c, and Rv1776c, are yet to be thoroughly characterized in transcriptional activity.

The specificity of TF binding and its effect on downstream gene expression have been shown in a plethora of studies to have dependencies on decoding the double helical 3-dimensional (3-D) structure of DNA (Rohs et al., 2009; Gordan et al., 2013; Inukai et al., 2017; Yella et al., 2018; Sarkar et al., 2021; Vanaja et al., 2021). There are several factors that contribute to DNA-TF recognition, including DNA shape and flexibility, each of which influences binding in a slightly different way depending on the circumstances (Yella et al., 2018; Chiu et al., 2022; Ghoshdastidar and Bansal, 2022). Deformation in DNA is essential for a wide range of biological processes, and these can be mediated by DNA surface topography, shape, and mechanical characteristics (Bansal et al., 2014; Kumar et al., 2016; Kumar and Bansal, 2017; Basu et al., 2022; Vanaja and Yella, 2022). In this way, the inherent structural features of DNA, such as its deformability, duplex integrity, curvature, groove form, and topography, are more reliable indicators of TF-binding site specificity in DNA than just the conventional string of nucleotides (Yella et al., 2018).

Potential binding sites for all TFs can be found in significant numbers across the genome of any organism, but unfortunately, only a handful of these putative sites function efficiently. The nucleotide sequence in the basic motif is often insufficient for characterizing a cognate binding site in the genome. Therefore, an integration of DNA sequence and shape characteristics is necessary to redefine the TF binding site (TFBS), as it offers a completely new avenue to study facets of TF binding, in a way that could be employed in a wide range of structural classes of TFs (Zhou et al., 2015). Our earlier research demonstrated three unique DNA structural tendencies in six nucleoid-associated proteins (NAPs) of *M. tuberculosis*, comprising EspR, MtHU, Lsr2, NapM, WhiB4, and mIHF, shedding light on the relationship between the strategy for recognizing NAPs and the NAP-bound architecture of DNA (Sarkar et al., 2021). In this study, we introduce a comprehensive computational strategy to analyze the structural basis of DNA binding events involving 21 TFs selected for widespread binding sites in the promoter regions of the *M. tuberculosis* H37Rv genome.

## Materials and methods

### Genomic sequence datasets of the target sites of virulence transcription factors

The TF binding site dataset was obtained from the MTB Network Portal (http://networks.systemsbiology.net/mtb/) (Turkarslan et al., 2015). We considered only those TFs that have more than 50 binding sites reported in the promoter regions (−150 to +70 of TSS), defined by Minch et al. (2015). These criteria helped us to analyze data with confidence, as based on our observation, TFs with less than 50 binding sites can result in noisy DNA structural profiles. The 21 TFs filtered by these criteria formed our promoter-binding dataset: Rv0022c, Rv0023, Rv0081, Rv0465c, Rv0602c, Rv0678, Rv0757, Rv0767c, Rv0967, Rv1033c, Rv1404, Rv1423, Rv1776c, Rv2034, Rv2989, Rv3133c, Rv3246c, Rv3574, Rv3681c, Rv3765c, and Rv3830c.

Additionally, we considered genome-wide binding events (significant ChIP-Seq peaks (*p* < 0.01) at promoter sites along with other genomic regions, i.e., non-promoters) of those TFs that have more than 50 binding sites. We referred to this dataset as an extended dataset in the manuscript hereafter. This extended dataset consists of 51 TFs after filtering. The 2001-nt (−1,000 to +1,000)-long sequence regions relative to the calculated ChIP-Seq center position (at 0) of the respective target binding sites have been retrieved from the whole genome of *M. tuberculosis* H37Rv. The total number of TFBS considered for the promoter-binding dataset for this study is reported in **Supplementary File 1**.

### Nucleotide composition analysis of the TF regions

−1,000 to +1,000 nucleotide long sequence with respect to 0 at the peak center were extracted for each TF. Furthermore, sequence composition analysis was performed by calculating parameters including k-mers occurrence frequency, motif periodicity, and nucleotide skewness. In-house Perl scripts were being used to calculate the AT/GC skewness. It defines the asymmetric nucleotide composition of the leading and lagging strands found in most bacteria.

### Estimation of sequence-based local and global structural profiles

Studies suggest that physical properties of DNA regulate the interplay of a TF and its cognate DNA binding site. DNA structural properties such as electrostatic potential (EP), helical twist (HelT), minor groove width (MGW), propeller twist (ProT), and roll were obtained using the R package DNAShapeR (Chiu et al., 2016). This software utilizes a pentameric sliding window technique to generate representations of the above-mentioned shape properties obtained from the Monte Carlo simulation experiments. It accepts nucleotide sequences or genomic coordinates as input.

The trinucleotide model, which includes the DNase I sensitivity model (Brukner et al., 1995) and the nucleosome positioning preference (NPP) model (Satchwell et al., 1986), was used to calculate the bendability or flexibility of the given DNA sequence (Sarkar et al., 2021). Over the last two decades, the involvement of intrinsic DNA curvature in biologically crucial activities like replication, recombination, transcription, and chromatin architecture has been reported. It has also been observed that bent DNA elements are frequently located near functionally critical locations like promoters and replication origins. Following the discovery of curvature in sequences with periodic A-tract repetitions, numerous theories based on the specific features of oligo(A) tracts attempted to explain DNA curvature. Given this, a few models were available, including: two sets of dinucleotide parameters utilizing extensive datasets and several techniques, namely, BMHT (from the gel mobility assay) (Bolshoy et al., 1991) and CS (derived from the crystal structure data) (Kanhere and Bansal, 2003). Bolshoy et al. proposed the BMHT model, which is based on DNA gel retardation experiments. The 16 roll and tilt angles were derived from the gel mobility assay data. While it favors the AA/TT step, it overlooks rare occurrences like Dlakic and Harrington’s atypical helical phasing sequences in addition to motifs of the GGGCCC form (Bansal et al., 1995). DNAShape and global bendability properties for 51 TFs (in extended dataset) were visualized as heatmap after *Z*-score normalization for each TFs.

### Statistical analysis

To check for statistical significance, three subset regions were selected, each with 100 values from averaged structural profiles. As estimated from the center of the ChIP-Seq data, the central region (CR) is positioned between −50 and +50 nucleotides; meanwhile, the upstream (UR) and the downstream region (DR) are placed between −251 and −151 and between +151 and +251 nucleotide positions, respectively. Groupwise comparison of UR *vs*. CR and CR *vs*. DR was conducted to test the statistical significance. This strategy allowed us to compare statistically significant DNA sequence-dependent structural properties at the TF binding site (CR) with the flanking regions (UR and DR). To facilitate comparisons across groups, the Wilcoxon rank sum test was employed, utilizing the center portion as the benchmark. Results were shown as heatmaps, and at *p* < 0.05 threshold, the test was assessed to be significant.

## Results

The disparity within sequence motif and shape preferences of several DNA-binding proteins in *M. tuberculosis* was reported earlier from our lab. In this report, a thorough analysis of the motif periodicity, skewness in DNA sequence, local DNA shape features, and global properties of the TF bound genomic regions was conducted. The twenty-one mycobacterial TFs present in our dataset are classified into various TF families and display distinct DNA-binding motifs. While most of the TFs contain Helix-turn-Helix (HTH) DNA-binding motifs, winged-HTH (wHTH) motifs are also evident. While Rv0022c and Rv0681c contain CXXXC and CXXC motifs, information of DNA-binding motifs for Rv0967 and Rv1033c is not available. Therefore, questioning the probable DNA binding mechanisms of these TFs is particularly important. Here, we conducted in-depth analysis of DNA sequence and DNA sequence-dependent biophysical properties that might influence the DNA binding of those TFs.

### Sequence composition and skewness of TF interacting genomic sites in the *M. tuberculosis* genome

**Figure 1:**
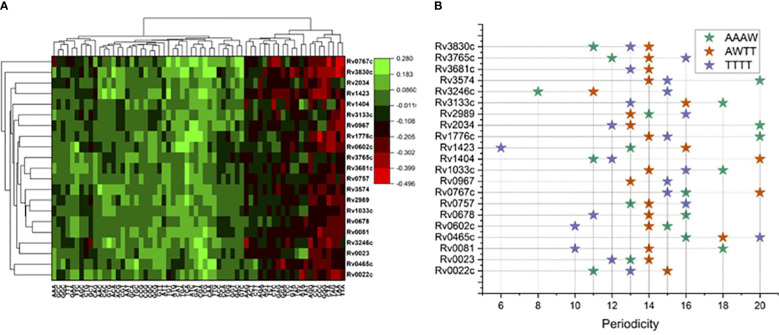
Sequence composition analysis of binding regions for 21 mycobacterial TFs. **(A)** Trimer enrichment at the TF binding sites at 101 long nucleotide regions relative to ChIP-Seq peaks at “0”. **(B)** Periodicity of four different tetrameric DNA motifs, color coded in the legend.

Relatively higher genomic GC composition of the *M. tuberculosis* (~65%) has preferences for few trinucleotides in its TF binding regions. Trinucleotide motif sequences like TTA, TAA, TAG, GGG, CCC, and CCT were depleted in the binding sites for most of the TFs included in this analysis, while CGA, TCG, TCA, ACA, and AAA and other trimeric motifs were comparatively enriched in the 101-nucleotide-long regions encompassing the TFBS (**Figure 1A**). Of note, few proteins have some unique preferences for specific trimers. Like Rv0767c, interaction sites were enriched for GAC and GTC while depleted for TAA and TTA. Such observations prompted us to investigate the preference of TFs for any nucleotide. Therefore, we sought to analyze the AT and GC skewness of the TF binding regions.

**Figure 2:**
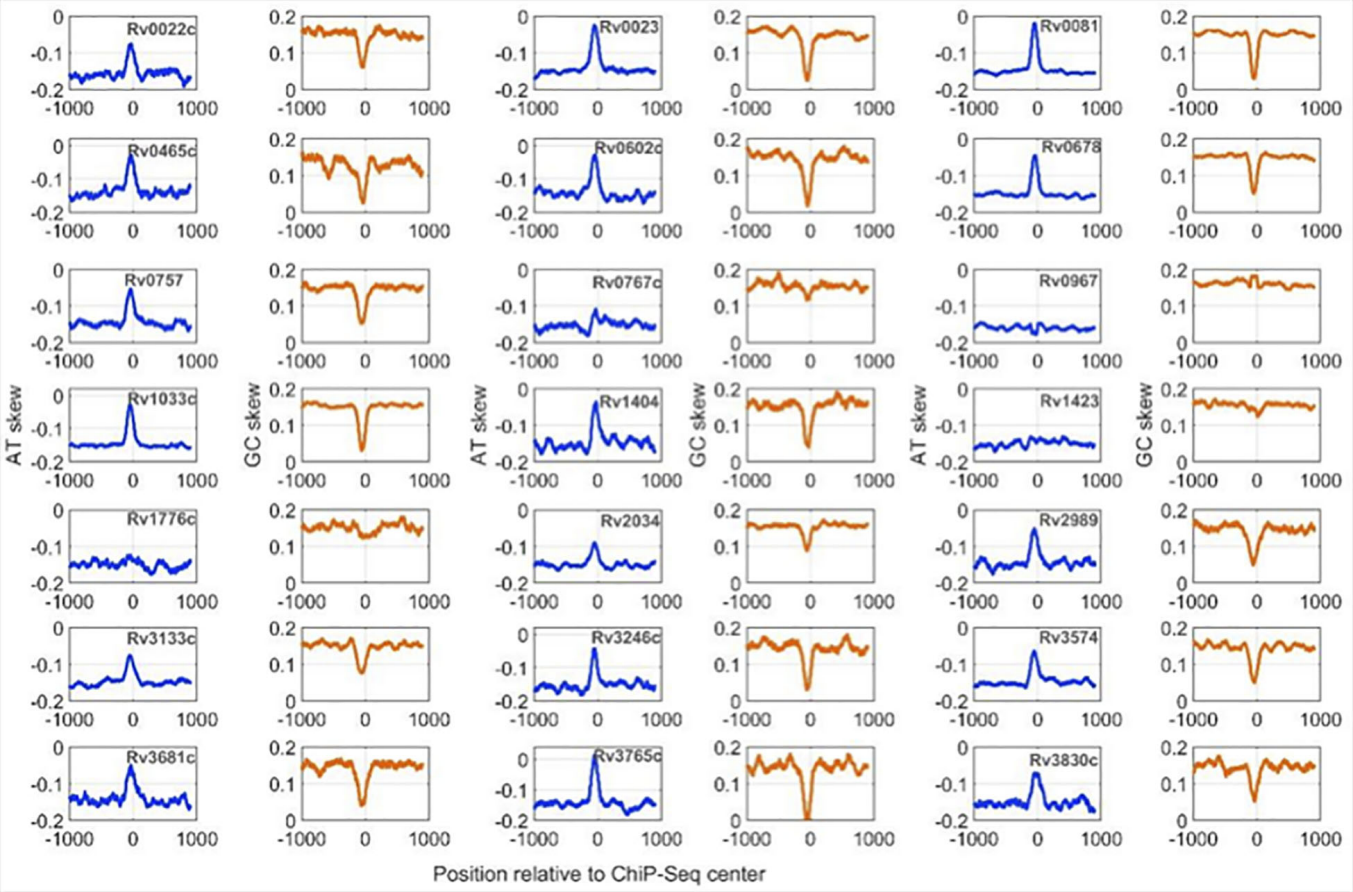
AT and GC skew profiles at 2001-nucleotide-long DNA regions encompassing ChIP-Seq centers. Profiles with a blue line are for AT-skew and the brown line corresponds to the GC-Skew.

Calculation of nucleotide distribution asymmetry in TF binding regions shows characteristic peaks for most of the mycobacterial TFs. For example, TFs like Rv0022c, Rv0023, and Rv0081 exhibit sharp peaks in AT-skew and downward peaks in GC-skew profiles (**Figure 2**). Rv0967, Rv1423, and Rv1776c, in contrast, depict a less AT-skew and higher GC-skew profile plots. Such variations in the profiles indicate that these TFs have strong preferences for A (adenine) nucleotides and less preferences for G (guanine) nucleotides. Moreover, high AT skewness and lower GC skewness at TFBS are reminiscent of regulatory regions, i.e., promoters of prokaryotic genomes. Additionally, our previous analysis of mycobacterial NAPs indicates similar AT and GC skew profiles in their DNA-binding regions (Sarkar et al., 2021). It was observed that NAPs preferring AT such as EspR, Lsr2, and mIHF exhibited prominent AT skew, while WhiB4, a GC preferring NAP, had characteristic GC skew in their binding regions.

Periodicity signals that were calculated for specific tetramers, i.e., “AAAW”, “AWTT”, and “TTTT”, which are known to influence local geometry of DNA molecule obtained interesting results. For example, for Rv0967 binding sites, 13 bp periodicity of TTTT and 15 bp periodicity of AAWT were evident. Considering the GC skewed binding regions of Rv0967 in mind, one tangible explanation for observing TTTT or AAWT periodicity could be the fact that GC-rich regions are linked to elevated physical stiffness of the DNA molecule (Tolstorukov et al., 2005) and often need a regular periodic signal of flexible k-mers (TTTT, AAWT, and AAAW) to introduce the necessary curvature. The lowest PSD values obtained for tetranucleotide motifs at the binding sites for 21 TFs were considered and plotted for visualization (**Figure 1B**).

### Local DNA shape and flexibility properties are evident in the TF binding sites in Mtb genome

**Figure 3:**
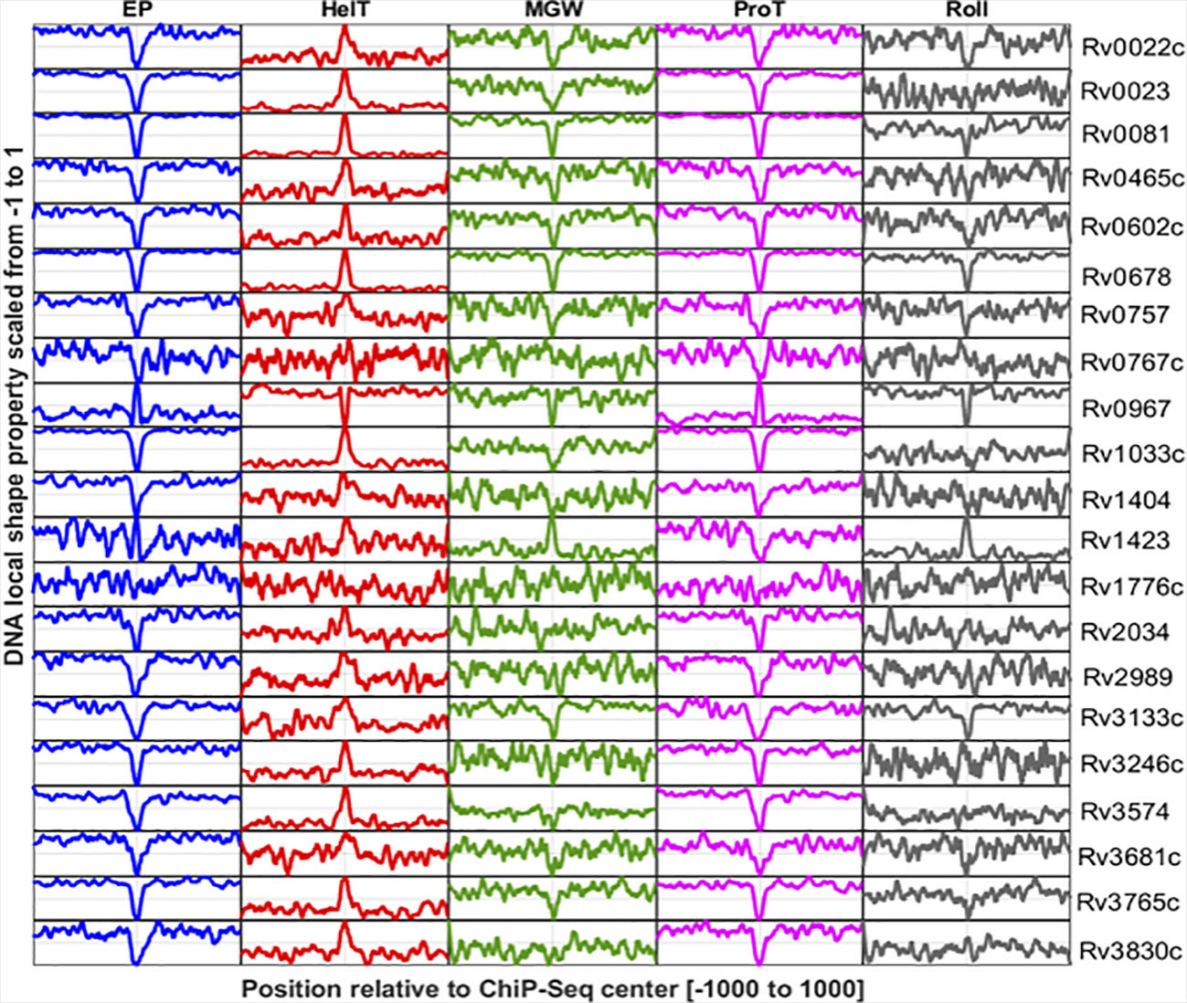
DNA shape profiles calculated for TF binding sites reported in promoter regions of 21 TFs. Columns indicate DNA shape features and rows indicate TFs. The shape profile calculated from the sequence is scaled from 0 to 1 and plotted. The *Y*-axis refers to the relative magnitude of the shape property while the *X*-axis corresponds to the genomic scale (−1,000 to +1,000 relative to the ChIP-Seq center of each TF).

DNA shape information embedded in the DNA sequence is characteristic of the regulatory genomic regions, and often manifested to underpin specific protein–DNA interactions (Yella et al., 2018). Here, we have explored the DNA shape parameters, i.e., MGW, Roll, ProT, HelT, and EP, in the TFBSs of the *M. tuberculosis* genome. Results indicate that base-pair parameters like MGW and ProT are characteristic of *M. tuberculosis* TFBS, while HelT and Roll, two base-pair step features, are less evident at those genomic regions (**Figure 3**). TFs encoded by the *M. tuberculosis* genome like Rv0022c, Rv0023, Rv0081, Rv0602, Rv0678, Rv0967, and Rv3133c have significantly lower MGW at their binding sites compared to the upstream (UR) and downstream regions (DR) (**Supplementary Figure S1**). In contrast, variation in MGW was not significant at the interaction sites for 10 TFs including Rv0757, Rv0767c, Rv1776c, and Rv2034. However, ProT and EP were evident at DNA binding sites for most of the TFs (**Figure 3**). TFs like Rv1423 and Rv0967 do not exhibit any significant changes in EP when compared to upstream regions of binding sites. Interestingly, unlike MGW, ProT, and EP, only three TFs, i.e., Rv0678, Rv0967, and Rv1423, have significantly different distributions of rolling angles at the core binding sites (CR) (**Supplementary Figure S1**).

DNA flexibility and curvature profiles of TFBS might be important for specific DNA recognition (Yella et al., 2018). DNA bending propensity for trinucleotide sequences obtained from high-throughput genomic studies provided a way to measure the flexibility of TFBS. Specifically, DNase I sensitivity and nucleosome positioning preferences (NPP) models were employed to quantify the DNA flexibility for binding regions of 21 mycobacterial TFs. The DNase I model suggests that Rv0022c, Rv0023, Rv0081, Rv0678, and other TFs (**Supplementary Figure S2**) have binding predilections towards the less flexible genomic regions. In contrast, bendability profiles of few TFs including Rv0602, Rv0757, Rv0767c, and Rv1033 have pronounced flexibility at their binding sites. Statistically significant differences in flexibility at TF binding regions compared to the flanking regions (UR and DR) imply its putative role in determining the TF binding events in mycobacterial genome. The NPP model provides insights into DNA bendability in terms of rotational preferences of DNA molecules towards minor and major grooves; it was observed that the DNA binding sites of Rv0022c, Rv0023, Rv0081, and Rv0678 are highly bendable (**Supplementary Figure S2**). For example, Rv1423 and few other TFs tend to interact at the less bendable regions (**Supplementary Figure S3**).

Intrinsic curvature of TF binding sites was also predicted using the BMHT models, and mean curvature values at each nucleotide position were plotted as curvature profiles. For most of the TFs, the binding sites are markedly curved in comparison to the flanking sequences. For example, Rv0022c, Rv0023, and Rv0081 tend to bind the intrinsically curved regions whereas binding sites of Rv1776c are less curved compared to the flanks (**Supplementary Figure S4**).

Additionally, in our extended dataset, DNA shape and flexibility profiles of genomic regions for 51 TFs were reported (detailed in the methodology). DNAShape and flexibility profiles for each of the 51 TFs were presented with pooled promoter and non-promoter DNA-binding regions (**Figures S5**-**S10**). We observed consistently lower propeller twist (ProT) angles near ChIP-Seq center regions, except for few proteins. For instance, Rv0967 and Rv1816 surprisingly show higher propeller twist angles near ChIP-Center regions compared to other TFs. The result might suggest an alternative mode of DNA binding mechanisms or warrants more research in this direction. Furthermore, we found that Rv0967 shows markedly opposite trends for ProT and EP at their promoter binding sites when compared to the other proteins (**Figure 3**). Some of the TFs in the extended dataset have highly bendable binding sites compared to the flanking regions. However, TFs with less bendable binding sites were also evident in *M. tuberculosis* genome (**Supplementary Figure S5**).

### EP and ProT are important properties of mycobacterial TF binding regions

**Figure 4:**
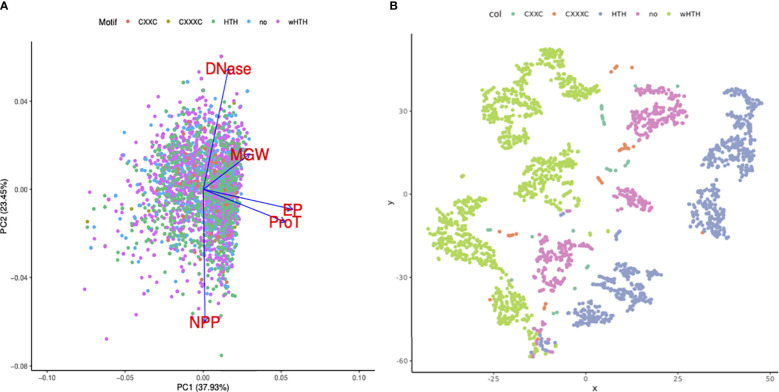
Importance of DNA structural features in TF binding regions. **(A)** PCA bi-plot shows the loading vectors in blue arrows. DNAShape properties like ProT and EP highly influence principal component 1. **(B)** t-SNE clustering of TF binding sites using DNA structural features, colored by the DNA-binding motifs present in the corresponding TFs. Green dots represent the sequences (transformed into shape features) that are bound by TFs with wHTH motifs. Sequences that are bound by TFs with wHTH and HTH clustered.

Sequence-dependent structural nuances in DNA molecules like shape, flexibility, and curvature are critical to the TFBS. This study largely focuses on elaborating the variations in the DNA structural properties of DNA binding sites for 21 TFs in the mycobacterial genome. Additionally, assuming a few of the structural features would be more important than the others, our objective was to identify the key features that might influence the TF binding event in the mycobacterial genome. Principal component analysis on the dataset suggested that propeller twist and the electrostatic potential of TFBS are major contributors towards PC1 (**Figure 4A**). PC1 and PC2 cumulatively explain ~61% of the variance in the dataset (**Figure 4B**).

Additionally, TF binding sequences are projected using the t-SNE algorithm into lower-dimension space. Minor groove width, electrostatic potential, and DNase I and NPP model predictions were used as features. DNA sequences that are bound by TFs with wHTH motif sequences, in blue color, clustered in the extreme right side on the *X* axis, while DNA sequences bound by TF with canonical HTH, in green color, clustered in the leftmost corner.

## Discussion

Public health is threatened by the emergence of *M. tuberculosis* strains resistant to multiple drugs. These strains are impervious to the most potent antibiotics, which makes treating tuberculosis more challenging and raises the likelihood of transmission. Hence, there is an imperative need to learn about the biology of Mtb. It is worth mentioning that advancements in the field of structural and computational biology have made significant contributions in this arena. By controlling the expression of genes involved in host–pathogen interactions, immune evasion, and bacterial persistence within host cells, TFs play a crucial role in the pathogenesis of tuberculosis (TB). When it comes to the binding of TFs in the Mtb genome, the significance of the DNA structure cannot be overstated. The binding affinity and specificity of TFs to their target DNA sequences are in part determined by the three-dimensional structure of DNA. In this study, we tried to explore the structural nuances of DNA, at the binding sites of TFs encoded by mycobacterial genome. The study highlights the presence of specific motif enrichment, local and global DNA sequence-dependent structures at the TFBS of mycobacterial genome. Also, this study highlights the need for a detailed re-evaluation of *in vivo* DNA-binding regions to augment the existing understanding of TF–DNA interactions.

The role of DNA-binding TFs in gene expression modulation is well established in current research. Numerous parameters, including sequence specificity and DNA deformation, are known to play key roles in TF–DNA interactions. While significant progress has been made in *in vitro* studies of several TF–DNA interactions, a detailed and distinct re-evaluation of *in vivo* DNA-binding regions is highly demanded to augment our existing understanding. Deciphering structural information through XRD, NMR, or Cryo-EM of protein and protein–DNA complexes is regarded as the gold standard in determining DNA binding mechanisms of any TFs. However, the crystal structure determination process is tedious, time-constrained, and of low throughput. Recent advances in molecular modeling and simulation techniques governed by biophysical principles, and integration of known protein–DNA complex structural information facilitate the genome-wide prediction of DNA structural nuances, directly from the DNA sequences. Moreover, DNA structural variations, at different scales, are evident in regulatory regions of the prokaryotic genome (Kumar and Bansal, 2012; Kumar et al., 2016; Kumar and Bansal, 2017). These conserved structural properties are often found conducive to crucial DNA–protein interaction events by modulating their specificity (Rohs et al., 2010). Estimation of DNA shape features, like minor groove width, electrostatic potential, and twisting angles; DNA flexibility from DNase I cutting experiments; and prediction of DNA curvature are few of the robustly validated models for explaining DNA–protein specificity across the domains of life. In line with this, to a significant extent, our study attempts to explore DNA structural nuances in the binding sites of 21 different TFs in *M. tuberculosis*. Moreover, this work is an effort to illustrate the possible role of evident DNA structural variation that might govern gene regulatory mechanisms modulated by the TFs.

Here, we delved into the trimeric nucleotide frequencies at the binding sites of 21 TFs encoded by the mycobacterial genome. Although *M. tuberculosis* is GC rich, few trinucleotide motifs were more enriched at the −50 to +50 region of TF binding sites. Moreover, periodic patterns of tetranucleotide motifs like “AAAW”, “AWTT”, and “TTTT”, which tend to deform the DNA molecule, were observed. Periodic cues in DNA sequences are well known and are of two types—the first being 3-bp periods, associated with the codon bias, i.e., corresponding amino acid implementation in protein coding regions, and the other being 10–11 bp or 10.5 bp (Herzel et al., 1999; Serizay and Ahringer, 2021). It was inferred that 10.5 bp supports DNA wrapping around nucleosomes, the formation of histone core, supercoiled structures of bacterial DNA, and nucleosome restriction signal within eukaryotes. While curved DNA segments intrinsically drive super-helical branching, it might facilitate the loop formation needed for TF–DNA interaction. Studies on bacterial and archaeal species documented conserved 10- to 11-bp and 10.5-bp periodic signals in their genomes, respectively (Lehmann et al., 2014). In addition, research on more than a thousand prokaryotic genomes suggests that a greater degree of structural plasticity in microbial genomes is facilitated by the presence of both strong and weak periodic signals (Mrazek, 2010). Additionally, a strong bias in AT and GC composition, i.e., skewness, was observed at the binding sites of multiple TFs. These results motivated us to look for enrichment of local and global DNA deformations at the TF binding sites.

Among the 21 TFs included in this study, 9 TFs, i.e., Rv0081, Rv0602c, Rv0678, Rv0757, Rv1404, Rv2034, Rv2989, Rv3246c, and Rv3765c, were reported to possess wHTH motifs. Rv0678, a member of the MarR-like family of transcriptional regulators, has been the subject of extensive research because of its role as a transcriptional repressor of the mmpS5-mmpL5 operon, which encodes an efflux pump capable of transporting anti-mycobacterial drugs such as bedaquiline and clofazimine. Recent research has shown that Rv0678 has mutations that reduce its binding activity to the promoter region of the mmpS5-mmpL5 operon (Gomez and McKinney, 2004; Andries et al., 2014). Computation of DNA-shape properties at the binding regions of these TFs suggests preferences towards narrower minor groove, and lower EP for DNA binding. Literature suggests that while the DNA recognition helix of wHTH motifs of TFs interact with the DNA major groove, the wings composed of beta sheets often contact the DNA backbone and minor grooves (Gajiwala and Burley, 2000). However, DNA regions with narrower minor groove width and lower EP features are not exclusive to the wHTH-containing TF binding sites; MGW narrowing was also evident for few other TFs in our dataset with the canonical HTH motif. According to the NPP model, binding sites of wHTH possessing TFs, except Rv3765c, tend to be highly bendable. In line with this, the BMHT-Dbyl model predicts that binding sites for those TFs are markedly curved compared to their flanking counterparts. It is intriguing to hypothesize that variation of DNA structures in the local scale (as measured by the DNA shape features), as well as in the global scale (estimated by the DNA bendability and curvature models), might influence the binding preferences of the TFs with wHTH motifs. DNA sequence with varying flexibility local shape properties is probably more important than shape-only features.

Rv0023 is an important transcriptional regulator having a canonical HTH DNA-binding motif, belonging to the XRE family of transcriptional regulators, which is among the most widespread regulatory elements present in bacteria (Rustad et al., 2014). A relevant study reported the role of Rv0023 in antimycobacterial drug tolerance such as isoniazide (INH) and ethionamide (ETO) (Gupta et al., 2020). In our study, it was found that Rv0023 has significantly lower MGW at its binding sites compared to the upstream and downstream regions. Additionally, the DNase I model suggests that Rv0023 has binding predilections towards the less flexible genomic regions. In contrast, the bendability profiles of Rv0023 have pronounced flexibility at its binding sites. It was observed that the DNA binding sites of Rv0023 are highly bendable. Rv1423 (WhiA) is a DNA-binding protein with probable involvement in *M. tuberculosis* cell division. Although it possesses the HTH motif for DNA binding, the explicit mechanism of its DNA binding is not yet clear (Knizewski and Ginalski, 2007). Here, our analysis hints that its DNA binding sites are less deformable, and intrinsically curved compared to the flanks. Additionally, we also observed widening of the minor groove, higher electrostatic potential, and lower propeller twist angles of nucleotides at the binding sites of Rv1423. However, additional experimental validation is required to support the significance of DNA deformations in its DNA binding events, observed at the Rv1423 DNA binding sites.

The 21 TFs encompass different TF families. TFs like Rv0022c and Rv3681c belong to the WhiB family, while Rv0767c, Rv1776c, Rv3133c, and Rv3574 contain the TetR domain. Although one study reported that DNA shape feature improves modeling of DNA-binding specificities across eukaryotic TF families (Yang et al., 2017), we observed no consistent DNAShape and bendability properties among members of same TF family in the *M. tuberculosis* genome. However, in our observation, DNAShape and flexibility properties are also evident in genome-wide binding sites of TFs, which are outside of the promoter region (**Supplementary Figure S10**). We observed considerable changes of propeller twist and electrostatic potential values for TFBS in the promoter region when compared to the non-promoter counterparts.

To summarize, this study described the unique shape markers seen at TF binding sites in *M. tuberculosis*. The results revealed various sequence-dependent DNA structural properties in the interaction sites of 21 TFs. Analysis of periodic properties in combination with DNA bending and shape features offered interpretations on the structural preferences of the putative binding preferences of those TFs. In line with this, research on *M. tuberculosis* virulent strains could aid in characterizing the structure of DNA double helix at the binding sites of TFs associated with virulence in stressful conditions. Studies are underway to check if the sequence-derived numeric features can predict the strength of DNA binding. In the future, genome-wide prediction of an accurate binding site along with binding affinity for any novel TF would be highly valuable to explain and modulate gene expression in pathogenic microorganisms.

## Data availability statement

The original contributions presented in the study are included in the article/**Supplementary Material**. Further inquiries can be directed to the corresponding authors.

## Author contributions

UD, VY and AK conceived the project. KO, AB and EA helped in data analysis, interpretation, and discussion. All authors contributed to the article and approved the submitted version.

## Supplementary material

Supplementary File 1

Supplementary Figure 1 Heatmap of p-values obtained from Wilcoxon test comparing DNA flexibility and five DNAShape properties at UR, CR and DR of TFBS. Details of the comparison methods are mentioned in methodology. For most of the TFs, ProT and EP values obtained for CR are significantly different from UR and DR regions.

Supplementary Figure 2 DNase I bendability profiles of 21 TF binding sites. Columns indicate DNase I sensitivity. X axis corresponds to the genomic scale (-1000 to +1000 relative to the ChIP seq centre of each TF). Y-axis units for each subplot is mentioned within the corresponding title of graphs. AU, arbitrary units.

Supplementary Figure 3 Nucleosomal Positioning Preference bendability profiles of 21 TF binding sites. X axis corresponds to the genomic scale (-1000 to +1000 relative to the ChIP seq centre of each TF). Y-axis units for each subplot is mentioned within the corresponding title of graphs. AU, arbitrary units.

Supplementary Figure 4 Intrinsic curvature profiles of 21 TF binding sites. X axis corresponds to the genomic scale (-500 to +500 relative to the ChIP seq centre of each TF). Y-axis units for each subplot is mentioned within the corresponding title of graphs. AU, arbitrary units.

Supplementary Figure 5 Minor groove width (MGW) at -200 to +200 flanking regions of ChIP-Seq peak sites reported in genome wide binding events for 51 TFs. Rows of the heatmap indicate 51 TFs, while columns indicate genomic positions with respect to ChIP-Seq centre. Promoter sets include all the bundling events reported by Minch et al. at -150 to +70 of TSS (promoter). Non-promoter binding sites indicate TF binding events outside of promoter regions (non_promoter).

Supplementary Figure 6 Propeller twist at -200 to +200 flanking regions of ChIP-Seq peak sites reported in genome wide binding events for 51 TFs. Rows of the heatmap indicate 51 TFs, while columns indicate genomic positions with respect to ChIP-Seq centre. Promoter sets include all of the bundling events reported by Minch et al. at -150 to +70 of TSS (promoter). Non-promoter binding sites indicate TF binding events outside of promoter regions (non_promoter).

Supplementary Figure 7 Electrostatic potential at -200 to +200 flanking regions of ChIP-Seq peak sites reported in genome wide binding events for 51 TFs. Rows of the heatmap indicate 51 TFs, while columns indicate genomic positions with respect to ChIP-Seq centre. Promoter sets include all the bundling events reported by Minch et al. at -150 to +70 of TSS (promoter). Non-promoter binding sites indicate TF binding events outside of promoter regions (non_promoter).

Supplementary Figure 8 DNase-I bendability at -200 to +200 flanking regions of ChIP-Seq peak sites reported in genome wide binding events for 51 TFs. Rows of the heatmap indicate 51 TFs, while columns indicate genomic positions with respect to ChIP-Seq centre. Promoter sets include all the binding events reported by Minch et al. at -150 to +70 of TSS (promoter in figure). Non-promoter binding sites indicate TF binding events outside of promoter regions (non_promoter in figure).

Supplementary Figure 9 Bendability calculated by NPP model at -200 to +200 flanking regions of ChIP-Seq peak sites reported in genome wide binding events for 51 TFs. Rows of the heatmap indicate 51 TFs, while columns indicate genomic positions with respect to ChIP-Seq centre. Promoter sets include all the bundling events reported by Minch et al. at -150 to +70 of TSS (promoter). Non-promoter binding sites indicate TF binding events outside of promoter regions (non_promoter).

Supplementary Figure 10 Comparison of DNA structural properties in TFBS in promoter and non-promoter regions. All the structural properties were evident in TFBS lying in those two genomic regions despite their difference in magnitude.
