# Supplementary material for: DNA structural properties of DNA binding sites for 21 transcription factors in the mycobacterial genome
Source: Front Cell Infect Microbiol. 2023 Jun 16;13:1147544. doi: 10.3389/fcimb.2023.1147544 (PMC10312376; doi:10.3389/fcimb.2023.1147544)
Supplement: Supplementary Figure 1 — Heatmap of p-values obtained from Wilcoxon test comparing DNA flexibility and five DNAShape properties at UR, CR and DR of TFBS. Details of the comparison methods are mentioned in methodology. For most of the TFs, ProT and EP values obtained for CR are significantly different from UR and DR regions. [file DataSheet_1.pdf]

Supplementary

DNA structural properties of DNA binding sites for 21 TFs in the mycobacterial genome

Upalabdha Dey[1], Kaushika Olymon[1], Anikesh Banik[1], Eshan Abbas[1], Venkata Rajesh Yella[2*], Aditya Kumar[1*]

[1]Department of Molecular Biology and Biotechnology, Tezpur University, Tezpur 784028, Assam, India

[2] Department of Biotechnology, Koneru Lakshmaiah Education Foundation, Guntur 522302, Andhra Pradesh, India

*Correspondence: Corresponding Authors

yvrajesh_bt@kluniversity.in

aditya@tezu.ernet.in

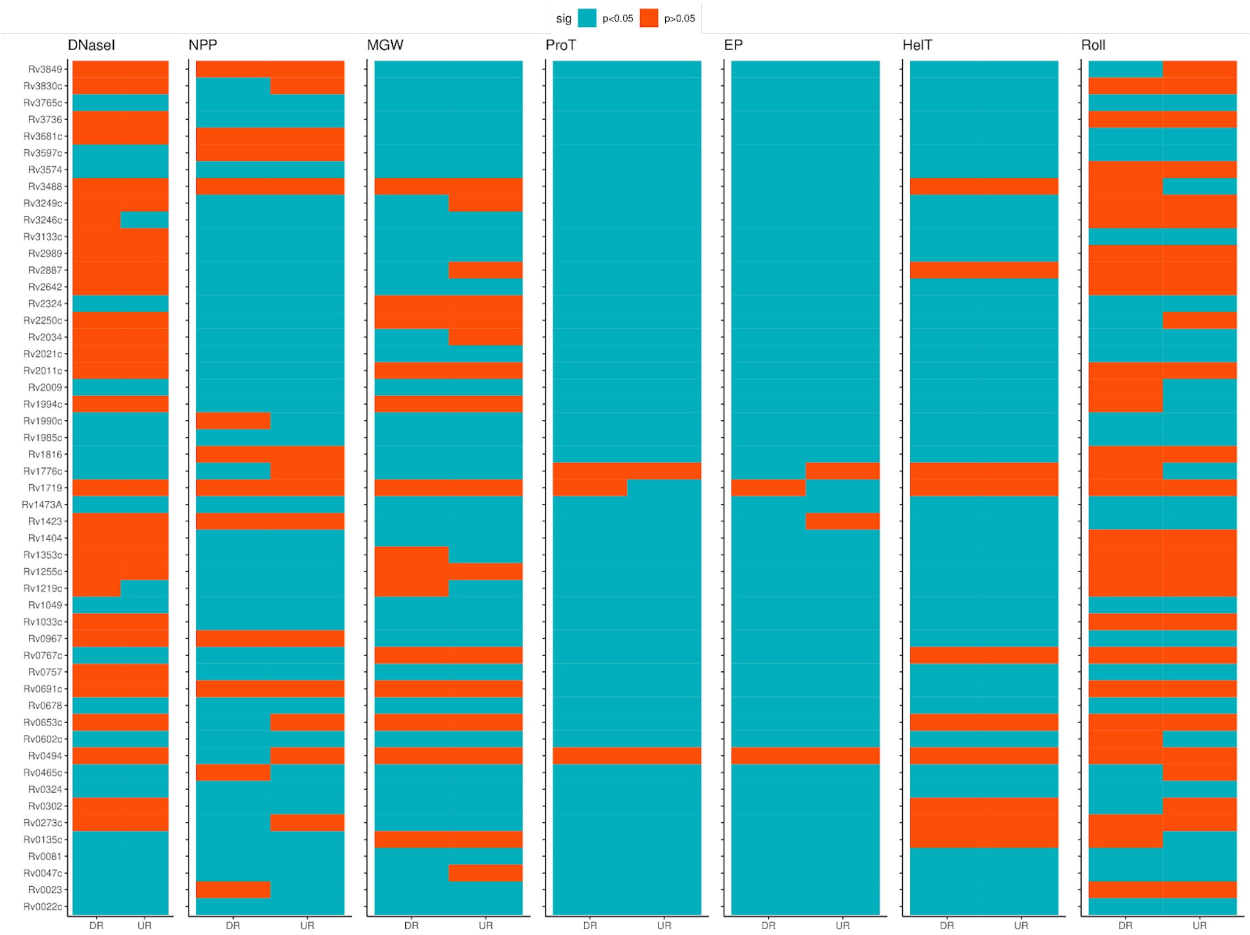

Figure S1: Heatmap of p-values obtained from Wilcoxon test comparing DNA flexibility and five DNAShape properties at UR, CR and DR of TFBS. Details of the comparison methods are mentioned in methodology. For most of the TFs, ProT and EP values obtained for CR are significantly different from UR and DR regions.

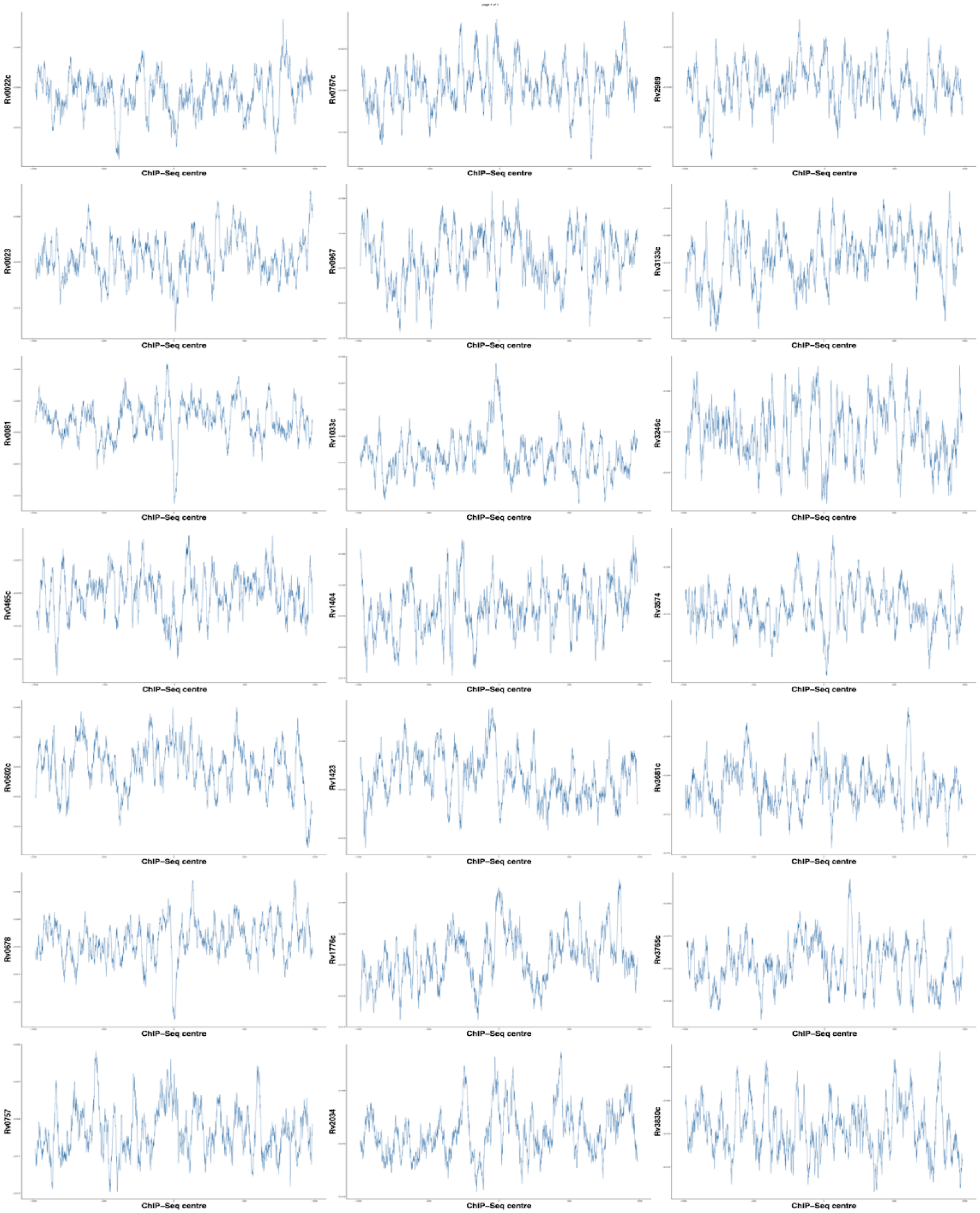

Figure S2: DNase I bendability profiles of 21 TF binding sites. Columns indicate DNase I sensitivity. X axis corresponds to the genomic scale (-1000 to +1000 relative to the ChIP seq

centre of each TF). Y-axis units for each subplot is mentioned within the corresponding title of graphs. AU, arbitrary units.

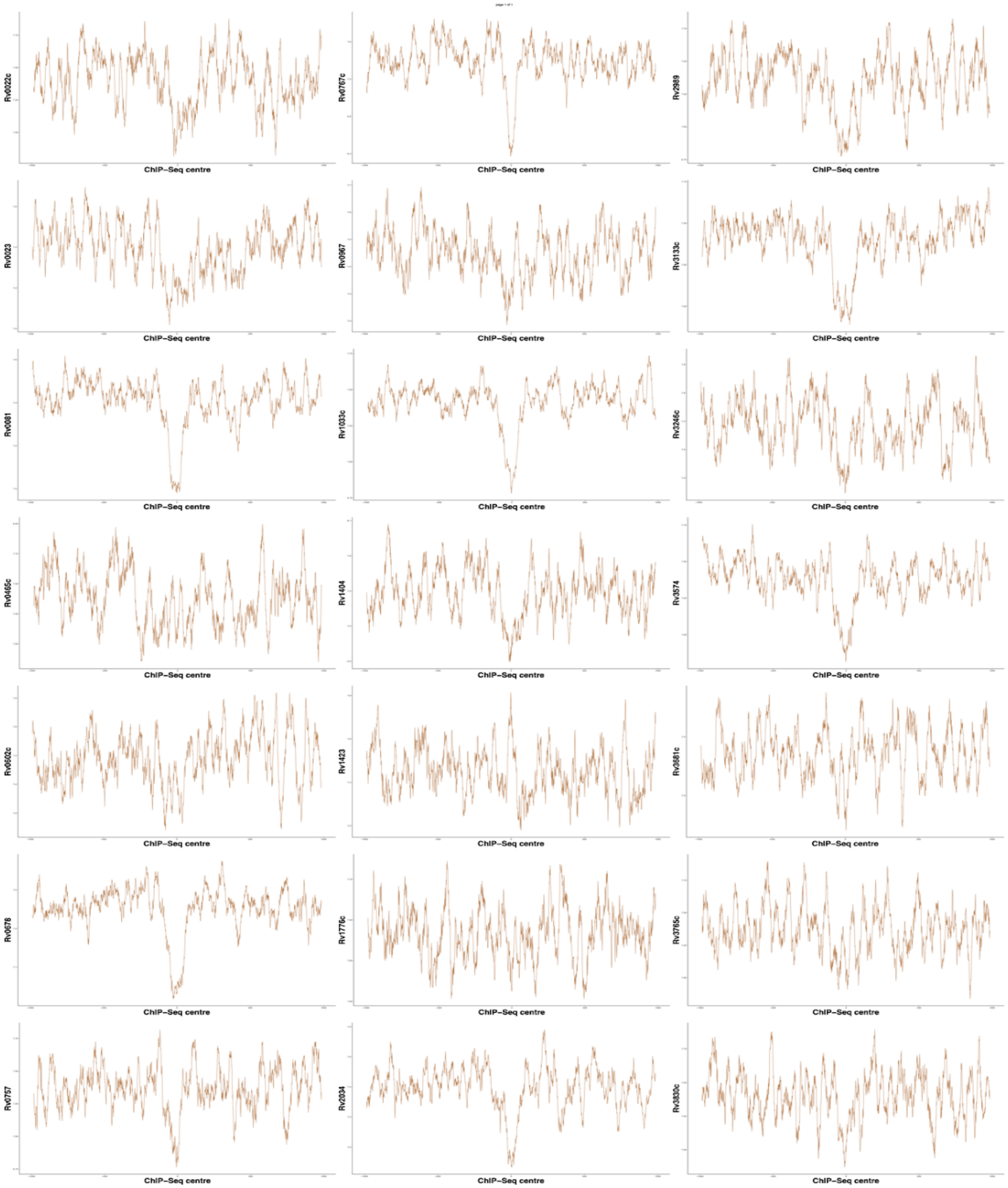

Figure S3: Nucleosomal Positioning Preference bendability profiles of 21 TF binding sites. X axis corresponds to the genomic scale (-1000 to +1000 relative to the ChIP seq centre of each

TF). Y-axis units for each subplot is mentioned within the corresponding title of graphs. AU, arbitrary units.

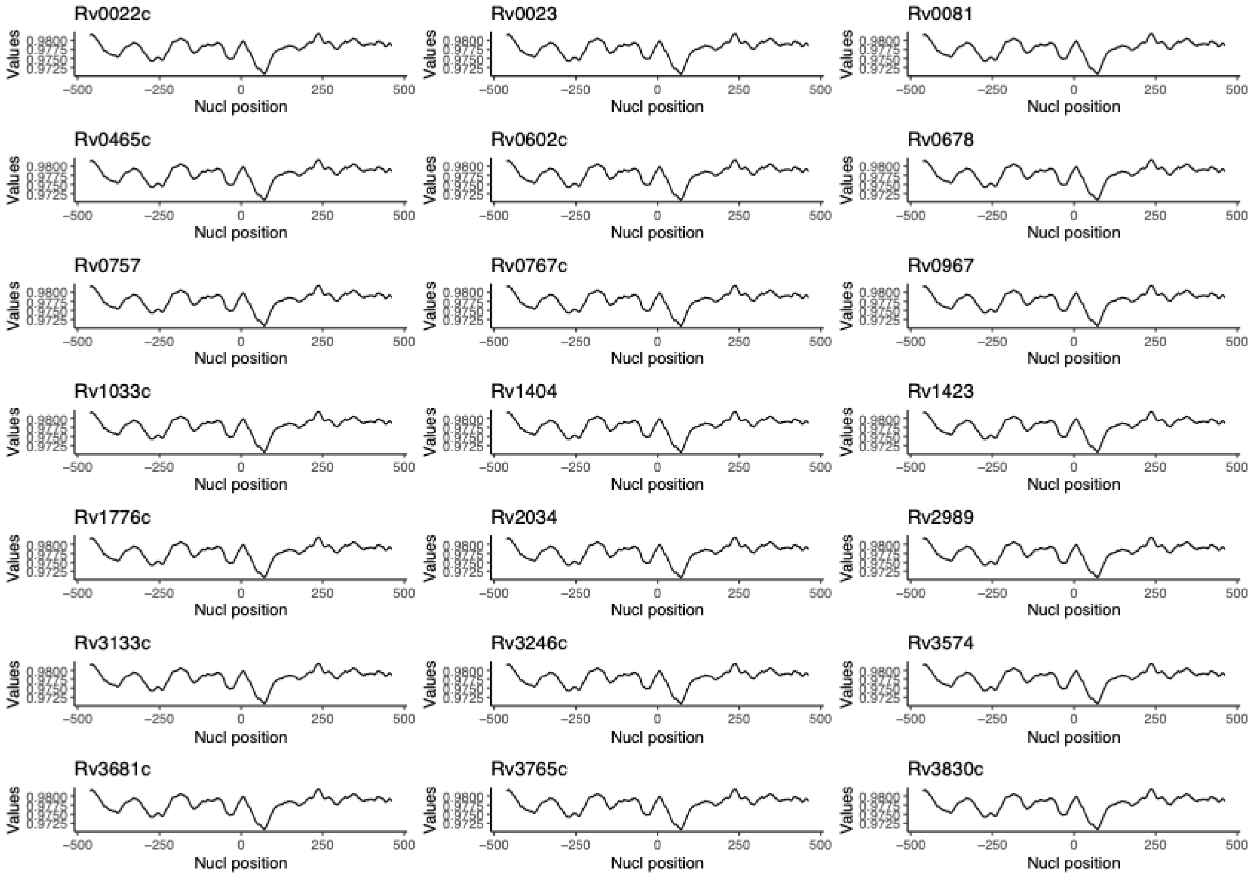

Figure S4: Intrinsic curvature profiles of 21 TF binding sites. X axis corresponds to the genomic scale (-500 to +500 relative to the ChIP seq centre of each TF). Y-axis units for each subplot is mentioned within the corresponding title of graphs. AU, arbitrary units.

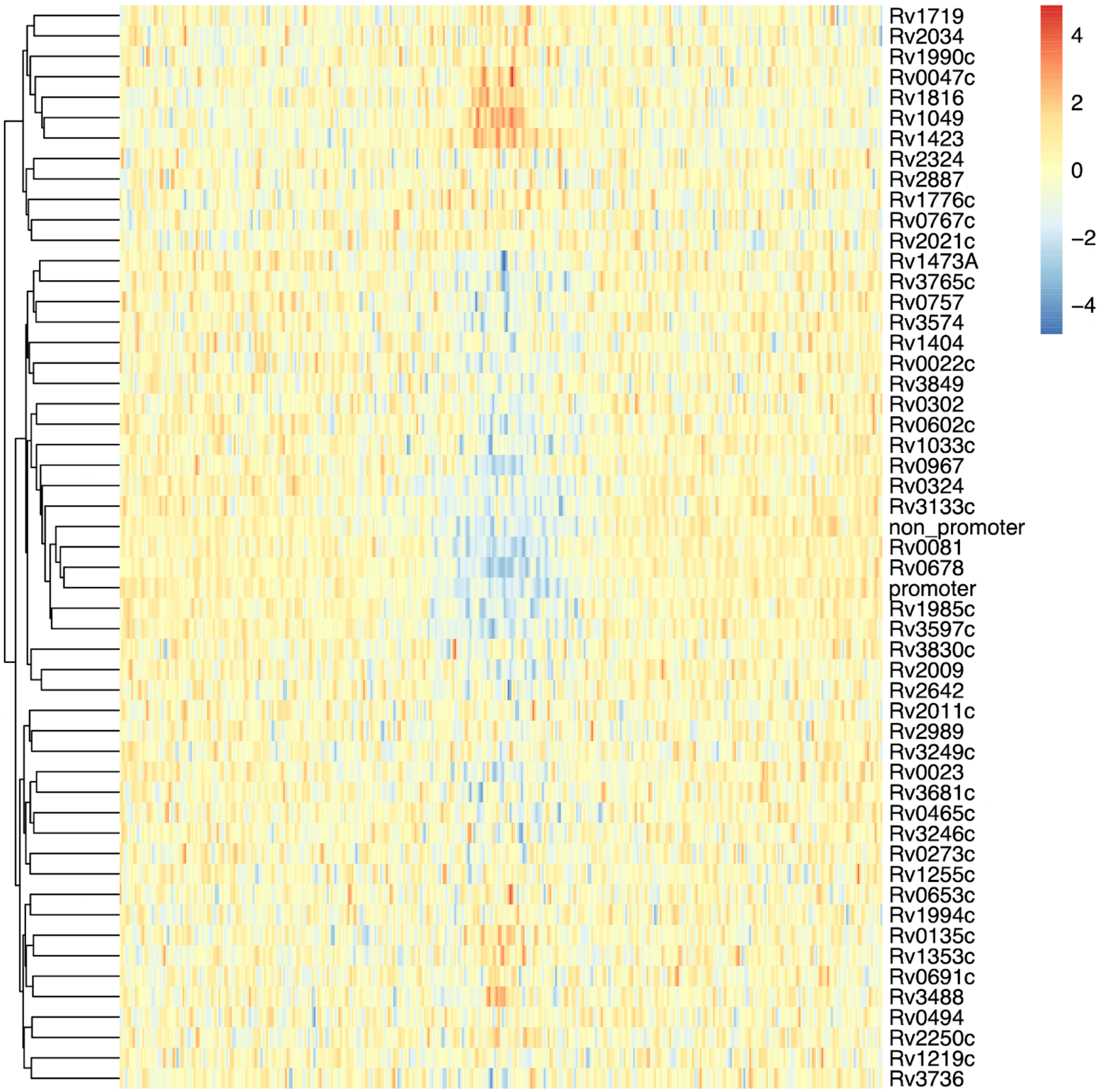

Figure S5: Minor groove width (MGW) at -200 to +200 flanking regions of ChIP-Seq peak sites reported in genome wide binding events for 51 TFs. Rows of the heatmap indicate 51 TFs, while columns indicate genomic positions with respect to ChIP-Seq centre. Promoter sets include all the bundling events reported by Minch et al. at -150 to +70 of TSS (promoter). Non-promoter binding sites indicate TF binding events outside of promoter regions (non_promoter).

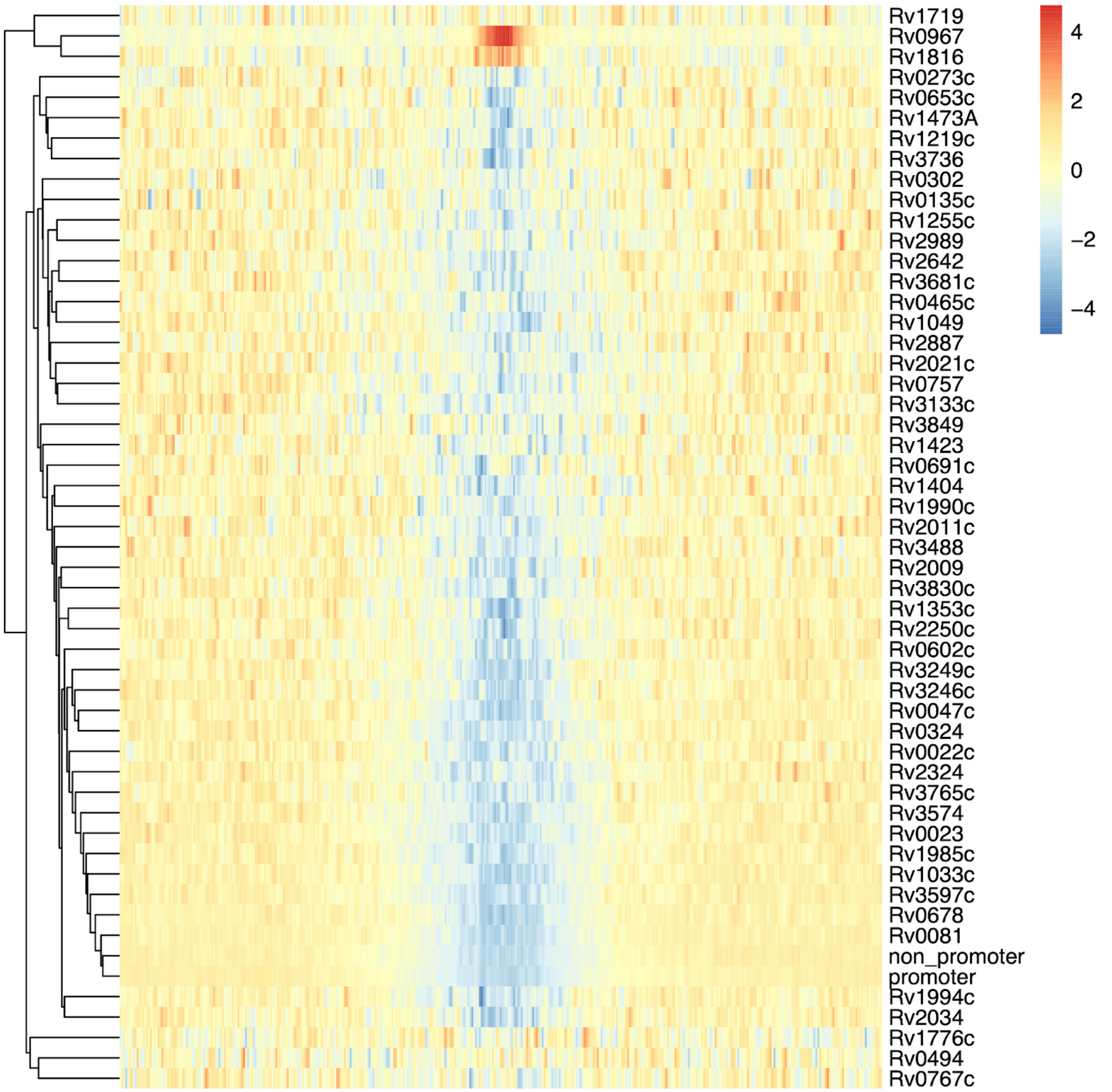

Figure S6: Propeller twist at -200 to +200 flanking regions of ChIP-Seq peak sites reported in genome wide binding events for 51 TFs. Rows of the heatmap indicate 51 TFs, while columns indicate genomic positions with respect to ChIP-Seq centre. Promoter sets include all of the bundling events reported by Minch et al. at -150 to +70 of TSS (promoter). Non-promoter binding sites indicate TF binding events outside of promoter regions (non_promoter).

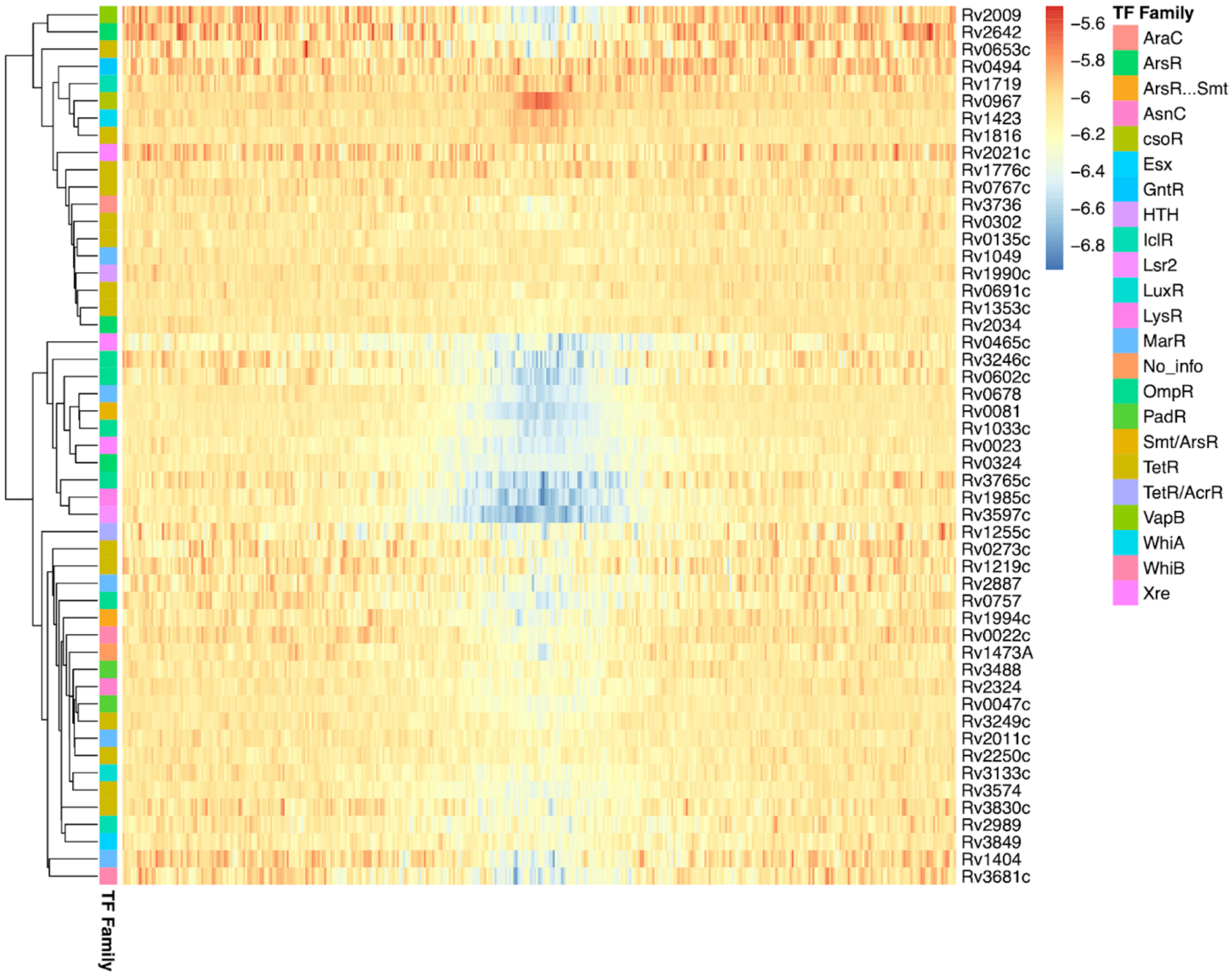

Figure S7: Electrostatic potential at -200 to +200 flanking regions of ChIP-Seq peak sites reported in genome wide binding events for 51 TFs. Rows of the heatmap indicate 51 TFs, while columns indicate genomic positions with respect to ChIP-Seq centre. Promoter sets include all the bundling events reported by Minch et al. at -150 to +70 of TSS (promoter). Non-promoter binding sites indicate TF binding events outside of promoter regions (non_promoter).

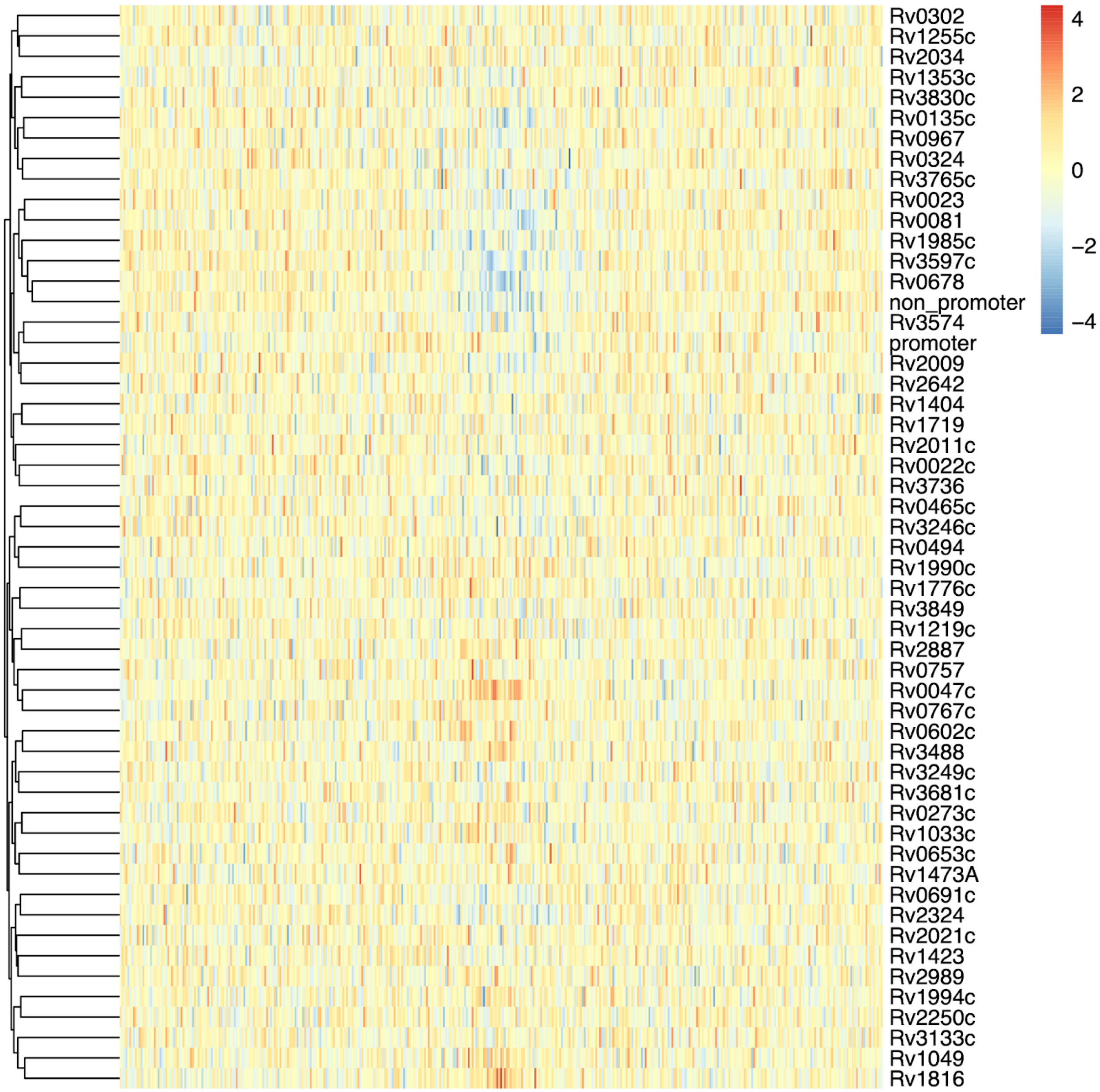

Figure S8: DNase-I bendability at -200 to +200 flanking regions of ChIP-Seq peak sites reported in genome wide binding events for 51 TFs. Rows of the heatmap indicate 51 TFs, while columns indicate genomic positions with respect to ChIP-Seq centre. Promoter sets include all the binding events reported by Minch et al. at -150 to +70 of TSS (promoter in figure). Non-promoter binding sites indicate TF binding events outside of promoter regions (non_promoter in figure).

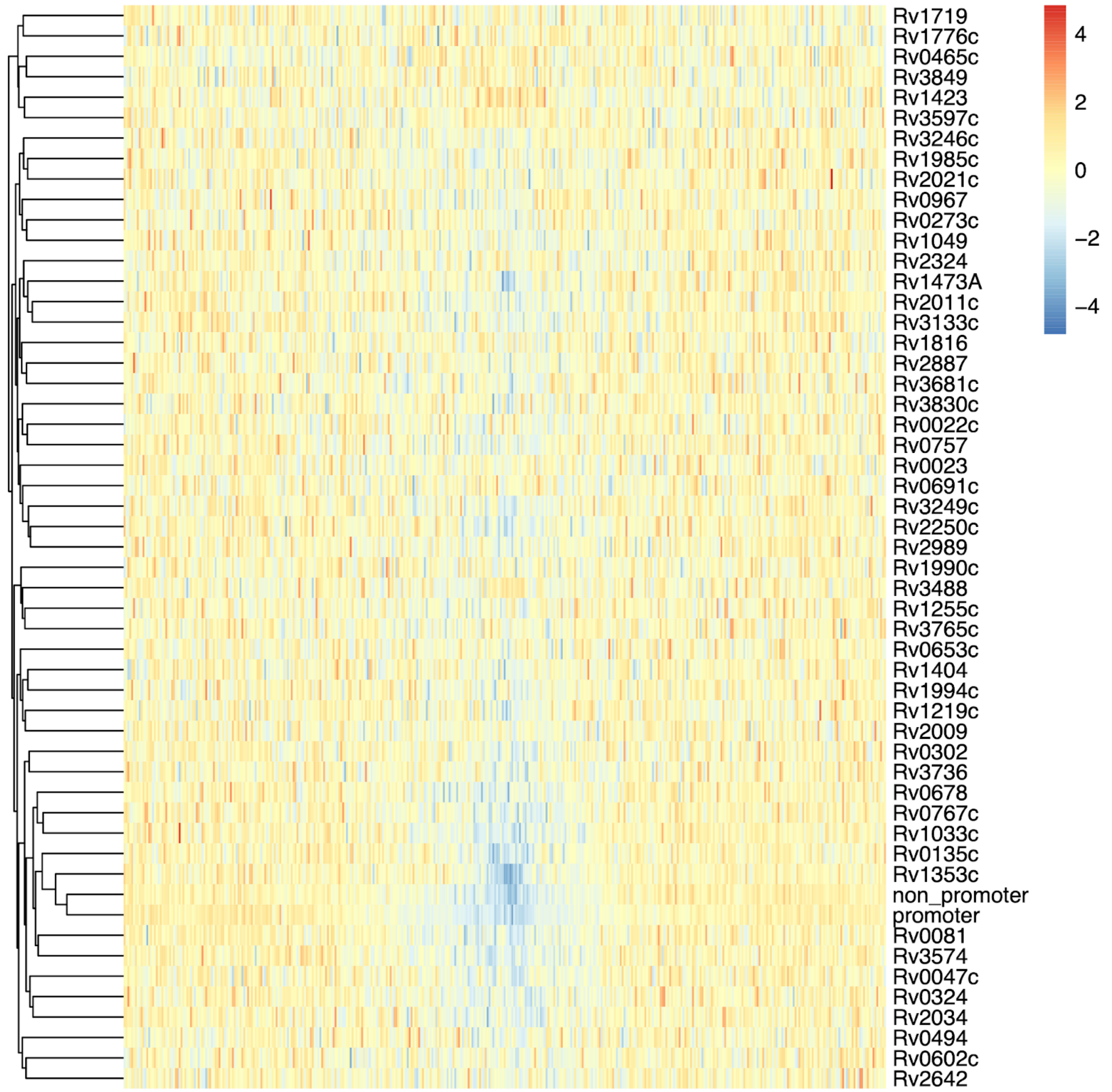

Figure S9: Bendability calculated by NPP model at -200 to +200 flanking regions of ChIP-Seq peak sites reported in genome wide binding events for 51 TFs. Rows of the heatmap indicate 51 TFs, while columns indicate genomic positions with respect to ChIP-Seq centre. Promoter sets include all the bundling events reported by Minch et al. at -150 to +70 of TSS (promoter). Non-promoter binding sites indicate TF binding events outside of promoter regions (non_promoter).

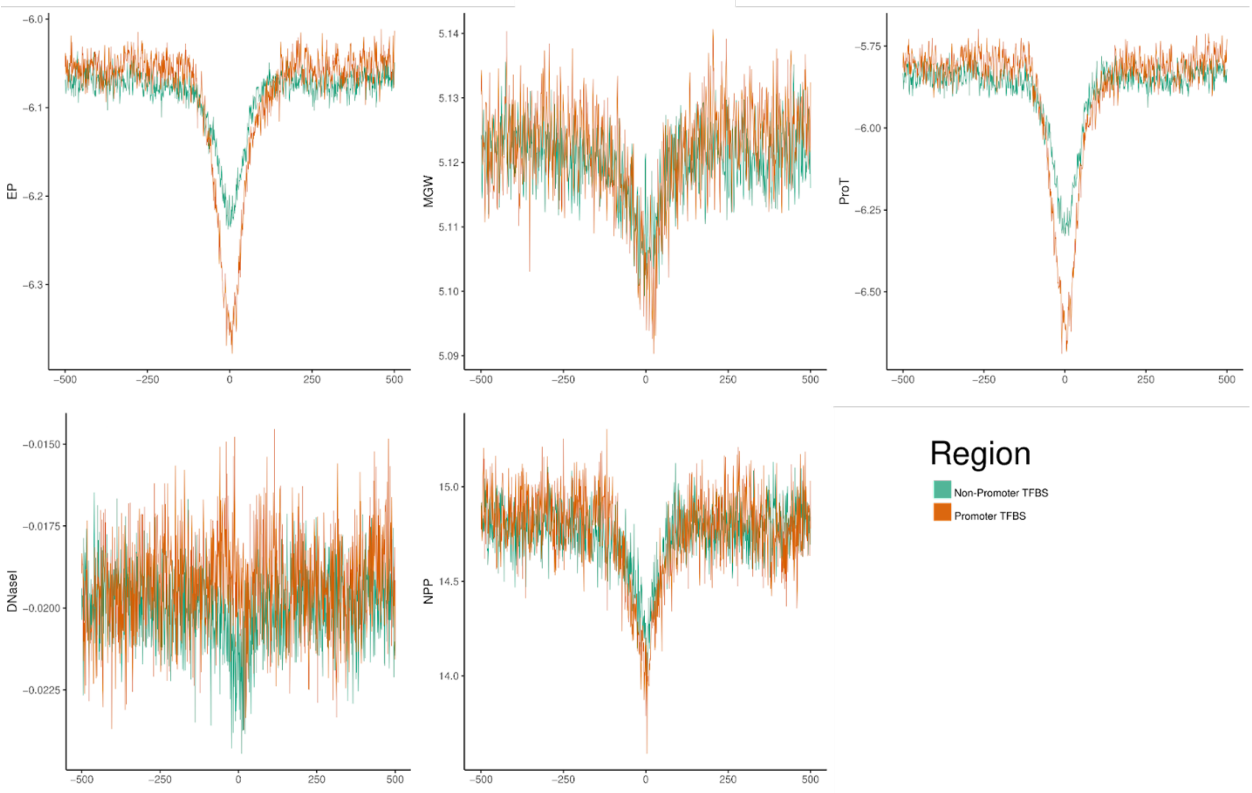

Figure S10: Comparison of DNA structural properties in TFBS in promoter and non-promoter regions. All the structural properties were evident in TFBS lying in those two genomic regions despite their difference in magnitude.
